# Influence of Feeding Omega-3 Polyunsaturated Fatty Acids to Broiler Breeders on Indices of Immunocompetence, Gastrointestinal, and Skeletal Development in Broiler Chickens

**DOI:** 10.3389/fvets.2021.653152

**Published:** 2021-06-28

**Authors:** Aizwarya Thanabalan, Elijah G. Kiarie

**Affiliations:** Department of Animal Biosciences, University of Guelph, Guelph, ON, Canada

**Keywords:** omega-3 polyunsaturated fatty acids, broiler breeder nutrition, broiler chicken, growth performance, immunocompetence, skeletal development

## Abstract

Modern broiler chickens are associated with rapid growth rates and superior feed efficiency. However, they are also susceptible to physiological and metabolic disorders (e.g., skin lesions, lameness, sudden death, enteric diseases, myopathies) that exert substantial economic losses to producers. This is further exacerbated by consumer pressure and mandated cessation of production practices such as indiscriminate use of antimicrobial growth promoters. Manipulation of broiler breeder (BB) nutrition and management can influence chick quality, robustness, and resilience to stressors in the production environment. The present review examines the role of feeding BB functional polyunsaturated omega-3 fatty acids (n-3 PUFA) and subsequent impact on the indices of immunocompetence, skeletal, and gastrointestinal (GIT) development in broiler chickens. Research in mammalian and avian models led evidence that perinatal feeding of long chain n-3 PUFA such as eicosapentaenoic acid (EPA) and docosahexaenoic acid (DHA) engender transgenerational effects through regulation of a variety of biological processes including development of vital organs such as skeleton, brain and GIT. It is shown that feeding poultry breeders n-3 PUFA decreases inflammatory states and enriches hatching eggs with n-3 PUFA and immunoglobulins. Further evidence also shows that after 15 days of incubation, chicken embryos preferentially utilize long chain n-3 PUFA-critical for optimal cell, tissues, and organ development. Enrichment of n-3 PUFA in newly hatchling tissues reduce proinflammatory eicosanoids with consequences of enhanced bone mineralization. Dietary n-3 PUFA also modulates breeder GIT microbiota with consequences of microbial colonization and succession in chicks. As well, research shows that feeding poultry breeders n-3 PUFA bolsters progeny immunocompetence through enhanced passive immunity and antibody titres against routine vaccination. In conclusion, it appears that chicks may benefit from the incorporation of n-3 PUFA in the breeder diets; however, little attention is paid to fatty acids composition in breeder nutrition. We also highlight gaps in knowledge and future research perspectives.

## Introduction

Poultry production has continued to grow since the end of world war II, accelerating exponentially in the last few decades (1). Due to the anticipated growth of the human population, it is estimated that the global consumption of poultry products' will climb to 145 million tons by 2029—accounting for 50% of the 12% increase in global meat consumption projected by 2029 (2). To meet production demands, modern broiler chickens have undergone intensive genetic selection, associated with rapid growth rates and superior feed efficiency, as well as short and low-cost production cycles. For example, a broiler chicken now reaches 2.44 kg at day 35 vs. 1.40 kg bird at day 35, 30 years ago (1). Continuous improvements in broiler production are primarily due to genetic selection for high performing biological and economic traits, nonetheless, albeit to a lesser degree advancement in nutrition, housing, health, and management practices have also contributed (3).

Intensive selection for growth has also significantly accelerated occurrence of metabolic disorders due to high nutrient intake, rapid growth, and high metabolic rate (4). Examples of specific metabolic issues include skeletal disorders from rapid growth, resulting in inadequate bone and tendon development, which are unable to support heavy broiler weight. Alongside skeletal issues, modern broiler chicks also present issues with immunocompetence and decreased resistance to pathogens. A comparison of immune responses between a 1957 vs. 2001 broiler chicken strain, showed 1957 strain had heavier bursas, spleen and cecal tonsils and higher antibodies (Immunoglobulin M and Immunoglobulin G) response post-challenge (5). Traditionally, antimicrobial growth promoters (AGP) have been used sub-therapeutically to ameliorate broiler chickens' low response immune system. However, due to consumer and regulatory pressures, the industry is moving toward a complete removal of AGP use. As a result, there are increasing concerns about the broiler's health; specifically the gastrointestinal tract (GIT), which may be compromised leading to increased susceptibility to enteritis. Furthermore, digestion insufficiencies and high feed intake lead to excessive undigested nutrients in the small intestine leading to bacterial overgrowth—ultimately causing inadequate growth (3, 6). In this sense, alternative methods to facilitate the establishment and maintenance of a healthy GIT, robust immune system and the overall broiler chicken productivity of chicks are of interest. The concept of developmental programming through maternal nutrition could present a means to blunt the aforementioned metabolic disorders in the offspring.

Egg fat is of considerable importance in the nutrition of the developing embryo as a source of energy and essential fatty acids (FA) such as linoleic (18:2 ω-6) and α-linolenic (18:3 ω-3) acids for synthesis of polyunsaturated fatty acids (PUFA)-rich membrane phospholipids and eicosanoids (7, 8). Substantial upregulation of cytosolic fatty acid-binding proteins (FABP) and preferential utilization of long-chain omega-3 PUFA (n-3 PUFA) have been observed in the later phases of embryo development (9). In fact, Saber et al. (10) reported a decrease in late-stage embryonic mortality in fertile eggs from BB fed flaxseed or fish oil enriched diets, possibly due to the improved incubation parameters due to PUFA availability. The long chain n-3 PUFA such as docosahexaenoic acid (22:6 n-3; DHA) and eicosapentaenoic acid (20:5 n-3; EPA) are critical for optimal cell, tissues, and organs structure and function. Thus, adequate supply of n-3 PUFA through the yolk could be pivotal in the development of metabolic and immune tissue functions with long-term implications on susceptibility to metabolic disorders in broiler chickens. This review will examine the role of functional n-3 PUFA in broiler breeder (BB) diets and characterize subsequent effects on indices of immunocompetence, skeletal and gastrointestinal development in broiler chickens. Current literature will be appraised on aspects of FA nutrition in BB, gaps in knowledge and areas of future research.

## Metabolic Issues in Broiler Chickens

Intensive genetic selection has resulted in a modern-day broiler chickens that can reach market weight faster with significantly less feed for unit body weight gain than it is predecessors. While this rapid growth has allowed for efficient poultry production, it has also resulted in metabolic issues.

### Musculoskeletal Disorders

Broiler chickens experience both leg weakness and leg lameness due to a rapid and disproportionate muscle accretion relative to the maturation of skeletal system (11). Leg weakness is classified as breakage, fractures, and lack of strength and is quantified by a reduced bone mineral content (BMC), bone mineral density (BMD) and poor growth (12). The tibia is the main focus of skeletal disorders such as leg weakness as it bears majority of the bird load and is affected the most during locomotion. In order to sustain rapid growth during the early weeks of life, the tibia increases circumferential and longitudinal growth, at the cost of reduced mineralization and high porosity, increasing fracture risk (13).

Leg lameness is characterized as an inability to walk or have a limp due to injuries or defects—examples include tibial dyschondroplasia and bacterial chondronecrosis with osteomyelitis (BCO) (12, 14). A broiler inability to support the body weight is often a result of tibial dyschondroplasia (TD), wherein the proliferating avascular, pre-hypertrophying growth plate cartilage does not change to hypertrophying cartilage (4). Therefore, it cannot be replaced by bone—ultimately resulting in an abnormal cartilage mass under the growth plate and bone weakness. Broiler BCO is also considered a common cause of lameness and is a bacterial infection of the femoral or tibial head, which can result in necrosis (14). The BCO can be viewed as an interface between a dysfunctional gut and skeletal deformities. The mechanical stress on the growth rates leads to microfractures in the cartilage layers, which paired with disruption of the gut from inflammation allows bacteria to translocate through the epithelial layer and into the femoral head (14). In conjunction with rapid growth, inflammation of the gut has been identified to play a negative role in bone development—a concern in AGP-free broilers.

### Gastrointestinal and Immunocompetence Issues

For optimal production, a broiler chick must have a healthy gut, which can be defined as a combination of digestive function, immune competence, and gut microbiota. Genetically, broilers have increased their feed intake as selection has aimed at growth performance. This growth is dependent on the efficient digestion, absorption and assimilation of dietary nutrients. As well, maximum digestion of available dietary nutrients is critical for disease prevention. The presence of undigested feed results in loading of substrates in the hindgut, encouraging proliferation of bacteria, causing dysregulation in the adaptive immune system due to changes in microbial metabolism leading to inflammation and potentially disease (15). In conjunction, the short life span of broiler chicken limits the potential for the complete development of an adaptive immune system. The adaptive immune system is estimated to take ~5–6 weeks post-hatch to fully develop, indicating a strong reliance on the innate immune system. Traditionally, AGPs were used to maintain a balance between “good” and “bad” bacteria, with a direct influence on ileal microbiota communities (16). However, as poultry production shifts away from AGP use to modulate the gut microbiota, questions on how to establish a well-balanced gut microbiota and well-established innate immune system rises.

## Broiler Breeder Management, Nutrition, and Omega-3 Fatty Acids

Optimizing broiler chicken production starts with chick quality at hatch, a characteristic that can be primarily attributed to BB nutrition and management. The nutrition of BB contributes to hatching egg fertility and hatchability, chick hatch weight and subsequent performance upon placement in grow-out facilities. All required and available nutrients for embryonic development are heavily dependent on the nutrients and components deposited in the egg by the hen (17). Modern BB nutrition is unique as it is critical to provide enough, but not excessive nutrients for maximum egg production (18). This is because BB have not only been intensively genetically selected for the favorable traits found in broilers, such as rapid and efficient growth but also for a successful lifetime of egg production (19). As such, the metabolic needs resulting from these characteristics are contradictory—BB possess the genetics for broiler chicken production rates requiring high feed intake; however, excess body weight leads to low egg production and egg size variability in a flock. Furthermore, as poultry production eradicates the usage of AGP, BB production must also consider ways to maintain a healthy gut to prevent intestinal and inflammatory disorders which could negatively affect productivity and performance. A compromise of the hen's intestinal health affects the ability to absorb nutrients, affecting the ability to produce eggs as well as the nutrients deposited in the yolk for embryogenesis. Lastly, a compromised intestinal tract in broiler breeders also presents the potential to transfer pathogenic bacteria such as *Escherichia coli, Salmonella enterica* and *Campylobacter* to progeny during egg formation and passage (20–22).

To meet their unique metabolic demands, BB nutrition is constantly changing with their life stages. The first limiting nutrient in these diets is energy for proper body maintenance, growth, and production demands (23–25). Energy requirements are met through the fortification with lipid sources such as vegetable oils and animal fat. In particular, long chain n-6 and n-3 PUFA are considered essential due to the inability of avian species to insert a double bond beyond Δ−9 carbon due to the lack of Δ-12 and−15 desaturases and must be provided through the diet (26). Current practices of feeding cereal and vegetable protein-based diets have been well-documented for their imbalance of n-6 to n-3 FA ratio (27). A high dietary n-6 to n-3 PUFA ratio is known to create a prothrombotic and proaggregatory physiological state, encouraging inflammation and disease pathogenesis (28). Enriching diets with n-3 PUFA decreases the competition between alpha-linolenic acid (n-3) and linoleic acid (n-6) for conversion and elongation to their active metabolites: eicosapentaenoic acid (EPA) and docosahexaenoic acid (DHA) and arachidonic acid (AA) and increases n-3 PUFA metabolites. Reducing the difference between n-6 and n-3 PUFA ratio is not only beneficial for BB production but also to the progeny.

The FA profile of BB eggs is reflective of the FA composition of the diet, and therefore can be enriched with n-3 PUFA for embryonic utilization (29, 30). Beneficial effects of feeding BB n-3 PUFA enriched diets on offspring immunity, gut microbiota, skeletal development, modulation of hepatic and cardiac function have been reported. Oxidation of FA provides 95% of the total energy needed for embryonic development, with a preference of incorporating PUFA into the tissues (31–33). Polyunsaturated fatty acids constitute most of the structural phospholipids in the cell membrane of the developing embryo, affecting immune cell FA composition as well as the FA profile of the gastrointestinal tract. The gastrointestinal tract acts as mucosa-associated lymphoid tissue, referred to as the GALT, responding against bacterial, viral, and parasitic antigens (34). Excessive feed in the gut due to broiler growth demands and short intestinal passage rate induces stress on the gut, causing inflammation and intestinal barrier leakage. Therefore, the increase of n-3 PUFA in chick tissues may be beneficial for a more controlled immune response (35). As previously mentioned, n-3 PUFA inclusion also affects BB microbiota, which is passed on from BB to newly hatched chicks and can affect broiler immune status and production. Lastly, modulation of inflammatory responses in broilers also influences adequate bone development, another major developmental issue in poultry production.

## Omega-3 Fatty Acids in Broiler Breeder Diets and the Effect on Progeny

### Inflammatory Mediators and Antibodies

Newly hatched chicks are exposed to a variety of stressors associated with routine husbandry practices, including vaccination, handling, heat stress, and feed change. Whether these stressors occur singly or simultaneously, they have the potential to stimulate the stress response, impair immunity and negatively affect bird growth and well-being. The acquired immune system of a broiler chicken takes ~4–6 weeks after the 10 days post-hatch period to fully develop (36). In this sense, there is a strong reliance on the innate immune system, and the yolk derived maternal antibodies for protection during the early life stage due to broiler's short life span (37). The protective role of maternal immunoglobulins (IgY and IgA) is of particular interest due to the precocial nature of chickens (38). These maternal antibodies are provided during the process of egg formation and continue to function in the hatchling until its own immune response can take over (38). Manipulation of the maternal diet not only influences the passive and innate immune system of broiler chicks but also subsequent development of the acquired immune system through subtle variations in the epigenetic regulation of immune gene expression (39).

Inflammation serves as a normal innate immune response to a challenge, particularly during the first week of life when the chick experiences environmental, nutritional, and developmental changes. However, excessive or uncontrolled inflammation can steer nutrients toward the acute phase response and cause decreased feed intake, muscle protein accretion and increase metabolic rate, ultimately resulting in poor production (40). Eicosanoids are lipid mediators of inflammation, produced through the cyclooxygenase (COX) and lipoxygenase (LOX) pathways—utilizing AA and EPA as substrates (41). Eicosanoids derived from AA are generally more potent, can increase the production of pro-inflammatory interleukins and may play a pathogenic role (42, 43). Inclusion of 3% fish oil, providing EPA and DHA, into maternal diets resulted in a significant increase in the production of EPA derived leukotriene B_5_ (LTB_5_) by thrombocytes, which is less pro-inflammatory than AA derived leukotriene B_4_ (LTB_4_), at day 7 and 14 in progeny compared to progeny from breeders fed conventional corn-soybean meal diets (44). The EPA and DHA competitively inhibit the metabolism of AA in the COX and LOX pathways, supressing the up-regulation of the genes for enzymes required for LTB_4_ production while EPA also acts as a substrate for LTB_5_ production (42). Incorporation of yolk omega-3 FA from enriched maternal diets into chick cell membrane during embryogenesis may result in a less potent inflammatory response to early life stressors.

Furthermore, the FA profile of immune organs in the progeny, specifically the spleen and bursa reflect the FA profile of the maternal diet; indicating incorporation during the developmental stage (44). A study examining effects of n-3 FA (DHA and EPA) inclusion from 1.5% replacement of the basal diet with fish oil blends in maternal diets reported the heaviest bursal weights upon hatch in chicks from EPA fed BB, potentially indicating an enhanced immune status due to the increase in B cell proliferation and maturation (45). Chicks hatched from BB fed diets enriched with 3.5% fish oil produced significantly less of AA derived eicosanoids, prostaglandin E_2_ (PGE_2_) and thromboxane A_2_, which are known for being pro-inflammatory and encouraging vasoconstriction and platelet aggregation, than their low n-3 PUFA dietary counterparts (46). Bullock et al. (47) reported a decrease in the pro-inflammatory regulatory cytokine interleukin–6 (IL-6) in both the liver and serum of chicks hatched from BB fed diets enriched with n-3 PUFA via 3.5% fish oil in the diet. As previously mentioned, n-6 and n-3 FA competition occurs in eicosanoid production by COX and LOX enzymes. By increasing preferential incorporation of n-3 FA into cell membrane embryonically, there is a shift toward EPA and DHA derived eicosanoids, accounting for the decrease in PGE_2_ as well as PGE_2_ induced IL-6 (48). Serum concentrations of IgG in hens fed diets with 5% linseed oil inclusion by replacement in the basal diet, as a source of n-3 PUFA, were significantly higher than other dietary treatments (49). Egg yolk IgY was also higher in eggs from n-3 PUFA enriched hens—both IgG and IgY have a role in inflammatory responses. As immunoglobulins in the hen are transferred to the chick to provide passive immunity, these results indicated the possibility for more controlled inflammatory responses in newly hatched chicks (49). The type of long-chain PUFA released in response to inflammatory stimuli is dependent on the cell membrane phospholipid PUFA content and therefore increased n-3 PUFA in BB diets is reflected in broiler chicks upon hatch and could act as a means of modulating inflammatory disease (50).

Transient immunosuppression caused by routine vaccination regimens in early phases of chick life (e.g., IBD, Marek's disease, coccidiosis, etc.) can increase broiler flock susceptibility to secondary bacterial infections. Moreover, incidences of vaccination failure are prevalent in the field resulting in chickens with inadequate antibody titer level development or are susceptible to a field disease outbreak following vaccine administration (51). Vaccination inadequacy is often associated with immunosuppression related non-cellular (antibody) and cellular components of the immune system not functioning properly (51, 52). There are numerous feedstuffs that have been shown to have immunomodulatory effects in young birds such as functional n-3 PUFA, yeast metabolites and probiotics (53–55). Dietary n-3 PUFA had a positive influence on humoral immunity in broiler chickens measured by antibody titers against Newcastle disease virus (NDV) compared to the control diet (56). Hens fed n-3 PUFA (fish or linseed oil, regardless of low, medium or high dose) had higher antibody titers upon challenge with bovine serum albumin compared to hens fed corn oil (57). It was recently demonstrated that feeding pullet breeders and/or their progeny n-3 PUFA (DHA and ALA) increased embryonic utilization of DHA and antibody titers for infectious bronchitis and new castle disease post-vaccination (9, 58). Fish oil modified eicosanoid metabolism and attenuated growth-depressing effects in an *Eimeria tenella* infection model (59).

### Gastrointestinal Microbiota Development

The gut microbiota is characterized by microbial community comprised of commensal, symbiotic, and pathogenic microorganisms with significant roles in the gastrointestinal development, resistance to non-specific infections and immune function (60). The intestinal microbiota has a specific role in forming a protective barrier in the GIT mucosa against pathogenic bacteria by reducing their adhesion to the mucosa (55). However, intestinal tract disturbances are a common issue in modern broilers due to their rapid growth rate and excessive feed intake resulting in a “leaky gut” and inflammation caused by dysbiosis (61, 62). Subtherapeutic use of AGP allowed for homeostasis of the microbiota by reducing the microbial load in the intestinal tract, preventing dysbiosis and increasing nutrient availability for the chick (60, 63–65). Dysbiosis of the microbiota leads to cascade effects resulting in inflammatory responses, reduction in nutrient digestion, and negatively affecting production (64, 66). Upon hatching, chicks display a highly dynamic and variable microbiota, as a result of maternal, hatchery, transportation, and rearing influences (67). Additionally, interactions between gut microbial community and host immune system stimulate GALT maturation, subsequently, the adaptive immune system and production long term (55, 68). Developmental programming could allow for colonization of some microbiota before hatch, as BB transfer gut microbiota to their offspring. This presents an opportunity to manipulate maternal diets toward beneficial microbiota (55). Recently published research mapping chick microbiota relative to maternal and egg microbiota suggests establishment and inheritance of microbiota from the hen as early as the fertilization period and during egg formation in the oviduct (69). The most significant determinant of gut microbiota profile is the diet which is influenced by factors like nutrient composition (fat, protein, fiber, etc.) and ingredients used (63, 70, 71).

While the relationship between n-3 FA and immunity is well-established, emerging research indicates n-3 FA and the gut microbiota share critical pathways of immune system activation and inhibition (72). It was reported that increased ingestion of n-3 FA resulted in a higher number of bacteria from the *Lactobacillus* group (72). However, another study only indicated minor changes in cecal *Lactobacillus* in broilers fed a diet with 2% fish oil relative to control and no difference in birds fed 5% fish oil (73). The *Lactobacillus* group is known functionally for enhancing intestinal barriers and is from the phyla Firmicutes (74). An increase in the relative abundance of Firmicutes in broiler chicken intestinal tract was negatively correlated with the expression of proinflammatory cytokine IL–6 (75). Firmicutes are also associated with the capacity for energy harvest and production of short-chain FA, particularly butyrate. This process reduces unused nutrients in the GIT while producing more usable energy for the chick (55, 76). Reduction of unused nutrients in the gut can facilitate homeostasis in the microbiota and potentially ameliorating some of the inflammatory intestinal metabolic issues seen in broilers.

Intestinal permeability (IP) serves as a measure of gut epithelial integrity (“leaky gut”) in relation to the passage of bacterial endotoxins, such as lipopolysaccharides (LPS) through the intestinal walls and into circulation (77). Lipopolysaccharide-binding protein (LBP) is a marker of metabolic endotoxemia, a measure of the innate inflammatory immune response caused by the increase of endotoxin bacteria which triggers a pro-inflammatory cascade effect releasing cytokines such as IL-6 (77, 78). Robertson et al. (77) reported a significant decrease in IP and LBP in offspring from *Fat-1* mice, indicating a suppressive effect on LPS-producing/pro-inflammatory bacteria. Metabolic endotoxemia may also be reduced by decreased LPS circulation due to the increase of gut barrier protecting bacteria (e.g., *Bifidobacterium)* a consequence of reduced n-6 to n-3 FA ratio (78). Interestingly, there were no differences in circulating cytokine between progeny despite their role in the inflammatory cascade involving LPS (77). Kaliannan et al. (78) reported gut microbiota influence on differential effects of tissue n-6 PUFA vs. n-3 PUFA on metabolic endotoxemia—particularly in tissues with high n-6 PUFA to n-3 PUFA ratio which retains pro-endotoxic bacteria. Enriching broiler chick tissue with maternal feeding of n-3 PUFA could present an opportunity to decrease harboring of pro-endotoxic bacteria, reducing inflammation, and increasing productivity. The inclusion of n-3 PUFA into BB diets could not only help the progeny but also help decrease the transfer of pathogenic bacteria from BB to broilers during egg formation and lay. As previously discussed, an inflammatory physiological state leads to a dysbiosis of the gut microbiota, allowing for pro-inflammatory bacteria presence to proliferate and subsequently transfer to the egg and outer shell. Through the reduction of inflammation and pro-inflammatory bacteria, there is the opportunity to decrease horizontal transfer and increase chick health upon hatch.

### Skeletal Development

As with other aspects discussed in this review, issues of skeletal development in broilers are a consequence of genetic selection for rapid growth rates and muscle deposition (79). Equally dramatic increases in the size, growth rate and structural integrity of the skeleton have not been selected for, leading to leg disorders such as leg weakness (characterized as fractures, breaks, and a lack of strength) and lameness in broilers (4, 14, 79, 80). These issues ultimately lead to unfavorable outcomes such as culling, poor production, and economic losses (79). While genetic selection plays the majority role in bone formation, environmental effects such as housing, and nutrition can also affect bone development. In humans, nutrient supply before birth and early adulthood plays an essential role in determining health outcomes such as bone mass into adulthood (81). *In utero* delivery of n-3 PUFA had the most significant positive effect on bone formation and was determinant of bone health in offspring during early developmental phase (82). In broilers, the concept of developmental programming through BB arises again as bone mineralization and development begins in early embryonic phase. Coupled with the knowledge that stimulation of the nutrition in the breeder affects progeny growth and metabolism, there is the potential to mitigate some skeletal issues in chicks (83, 84).

Currently, studies have shown a positive association of n-3 PUFA in optimizing bone growth and mass. Lau et al. (85) reported a favorable association between n-3 PUFA and bone formation biomarkers in *Fat-1* transgenic mice, which are capable of producing n-3 PUFA from n-6 PUFA. The FA composition of the femur was increased, specifically with ALA, DHA, and EPA—this coincides with previous reports of bone FA profiles in tibial cortical bone of chicks reflecting their n-3 PUFA enriched diets (85, 86). Similar results were seen in offspring of female rats fed either ALA (flaxseed oil) or ALA + DHA (DHASCO oil) 5 weeks before mating; offspring were maintained on maternal diets—indicating the ability to manipulate bone FA embryonically (87). Lau et al. (82) presents a comprehensive look at FA profiles of the bone relative to enriched diets.

Bone mineral content (BMC), a widely used marker of bone health, had a significantly positive correlation with either DHA or EPA in *Fat-1* mice (85). Several other feeding studies reported the same positive correlation between BMC and n-3 PUFA in growing animals (88–90). The BMC of the tibia in growing Japanese quail was also the highest in n-3 PUFA supplemented birds relative to control (91). While several studies report positive effects, there are other reports of no effects of n-3 PUFA inclusion on BMC in both mice and avian species (92, 93). A study feeding pullet breeders either DHA, ALA or a standard control diet reported an increase in tibial ash weight and percentage increases in the offspring produced from 1% DHA fed mothers (9). It may be possible to apply these findings in BB nutrition to fortify bone strength in broiler chicks. Bone mineral density (BMD) reflects the bone mineral content per unit volume of bone material significantly affecting the bone strength—this biomarker is of interest as increased bone strength could provide more support to the rapid growth of broilers. Rats fed diets with 40 g/kg n-3 PUFA displayed a 5.2% increase in femur BMD relative to control (94). In conjunction, bone biomechanical response is also an indicator of bone strength. The humeri of laying hens fed 10% flaxseed oil diets (supplying ALA) displayed a greater BMD than those from n-6 FA fed birds (95). The authors also reported stronger, tougher and stiffer bones as measured through bone-breaking strength in tibia from n-3 PUFA group (95). These results coincided with reports of an increase in tibial bone breaking strength in growing Japanese quail fed 2% fish oil and linolenic supplemented diets (96). Liu et al. (91) study also reported a higher relative shear force response (29.3% n-3 PUFA group vs. 16.6% control group) and stress response in the tibia of growing quail. Lastly, *Fat-1* progeny of *Fat-1* one mice reported a higher BMD at week 5 (growing stage) as well as an increase in tibial bone strength as the mice aged, as tested by ultimate load, stiffness and yield points (97). Feeding ALA and DHA to pullet breeders and pullets supported cortical development at sexual maturity (18-wk of age) (98). In broilers, the tibia is the main load bearing site and is most affected during locomotion and lameness; therefore, positive correlations between n-3 PUFA and increased bone strength could help in reducing current skeletal issues. It is interesting to note that studies which found no effect of n-3 PUFA on bone qualities were generally in older animals, indicating the importance of bone formation at early stages of development.

There are several hypothesized mechanisms relating n-3 PUFA and bone health such as an increase in calcium absorption in the small intestine, modulation of bone marrow cells and attenuating mediators of osteoclastogenesis. Studies feeding growing rats n-3 PUFA, particularly DHA, displayed an increase in calcium balance and small intestine absorption—indicating an increased availability of calcium and therefore potentially increasing incorporation into the bone matrix (82). Another proposed mechanism of action involving n-3 PUFA and bone development involves bone marrow which contains mesenchymal stem cells that can differentiate into osteoblasts (bone-forming cells) and adipocytes—stipulating that the higher number of bone marrow cells available, the higher the potential for osteoblastogenesis (99). Rats fed DHA during early post-weaning displayed a 2-fold increase in bone marrow cell numbers relative to their n-6 PUFA fed counterparts (100). Dietary n-3 PUFA also enhances the translocation of the stem cells into the osteoblastic lines by enhancing transcription factors (101). Osteoclastogenesis is the process wherein bone-resorbing cells known as osteoclasts are formed. A triad of proteins regulates the process, and this includes nuclear factor β ligand RANKL, the receptor RANK and the decoy receptor osteoprotegerin (OPG) (102). The main regulatory cytokine involved in this process is the AA derived lipid mediator prostaglandin E2 (PGE_2_) and acts to stimulate RANKL and RANK, leading to maturation of osteoclast precursors into activated osteoclasts. Mouse bone marrow stem cells which were exposed to control, AA or EPA treatment showed the highest production of PGE_2_ in the AA group (103). Furthermore, the EPA group had an inhibitory effect on PGE_2_ production, which was related to lower COX-2 gene expression, the pathway which produces PGE_2_ (103). Offspring of n-3 PUFA fed mice also exhibited a significant decrease in the number of resorptive osteoclast cells, due to the downregulation of RANKL mRNA (81). As with many of the results presented in the literature review, research indicates that the reduction in dietary n-6 PUFA to n-3 PUFA ratio has the potential to enhance beneficial effects; in this case, bone formation (104). Decreasing the n-6 PUFA to n-3 PUFA ratio results in the decrease of proinflammatory n-6 PUFA derived cytokines such as IL-6 (95). Cytokines IL- 6 and PGE_2_ induce each other's production and prompt osteoclastogenesis through inhibiting OPG production and upregulating RANK production, indicating an inflamed state could perpetuate bone loss and weakening (91). Tong et al. (105) explored the relationship between inflammation and bone metabolism in broilers through an LPS challenge. LPS challenged birds had shorter tibial length and reduced tibial weight relative to control birds, as well as increased levels of pro-inflammatory AA, derived cytokines (IL-6, TNF α) and RANKL and decreased OPG levels (105). Cytokine IL-6 also decreased the height and proportion of the tibial growth plate, suppressing bone growth. Factors associated with osteoclastogenesis were also responsible for tibial dysplasia in chickens due to the decrease in vascular density (105).

The last concept we will discuss involving skeletal metabolic issues in broilers is lameness caused by bacterial chondronecrosis osteomyelitis (BCO). BCO is characterized as an infection in rapidly growing bones attributed to repeated mechanical stress (14). As previously mentioned, broiler chicks generally have underdeveloped immune systems and are often in state of stress during early development. During this immunosuppressed state, bacteria from the gut microbiome translocate across the intestinal epithelium into the circulation and spreads hematogenously, forming bacterial foci which sequesters within the infected bone at the growth plate and adjacent metaphysis where antibiotics and immune system cells cannot reach (14). While there is no research examining the relationship between BCO, inflammation and n-3 PUFA, we postulate that n-3 PUFA inclusion into diets could ameliorate some of the effects of BCO by decreasing the inflammatory intestinal state and thereby reducing bacterial translocation.

## Concluding Remarks and Future Directions

**Figure 1:**
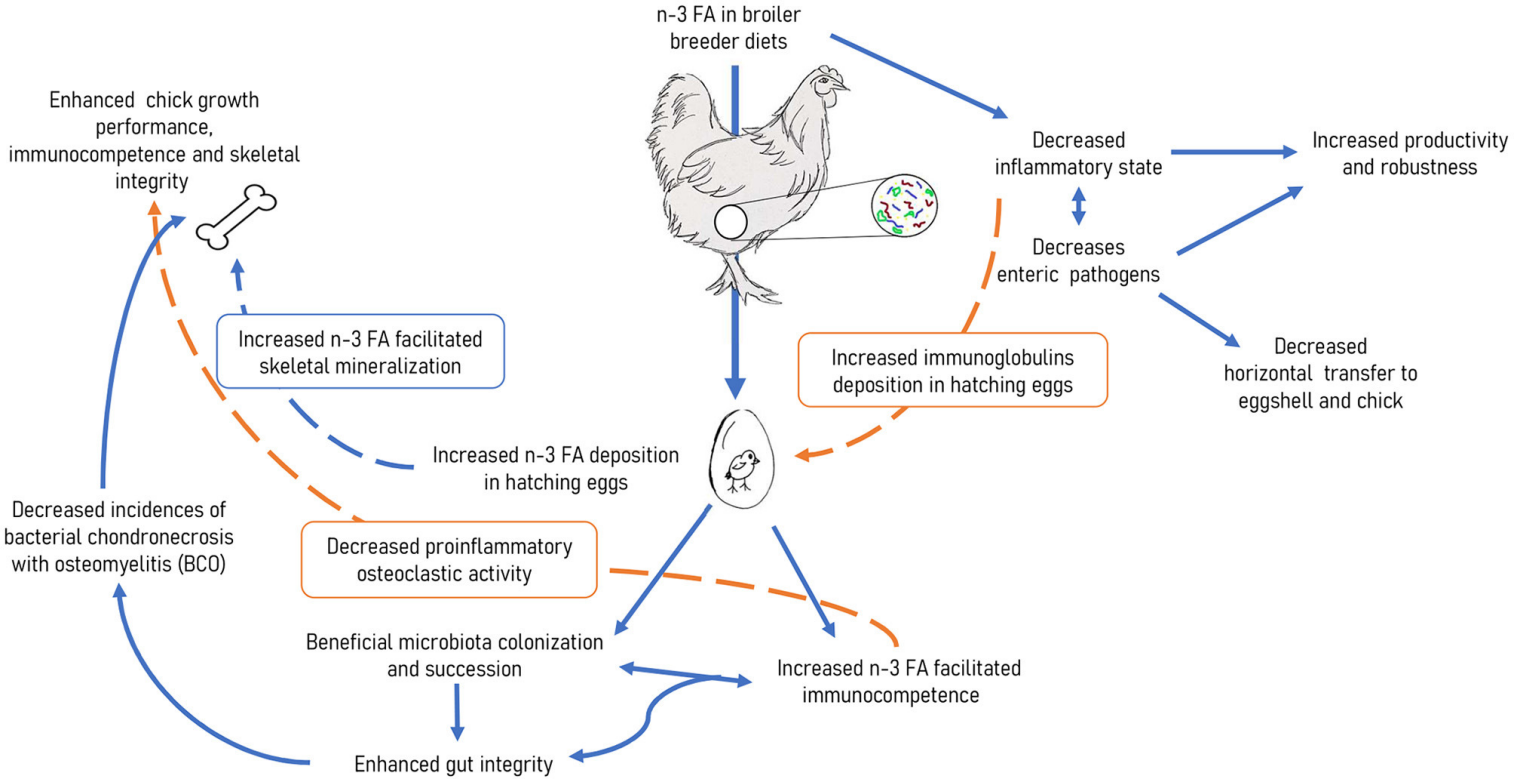
Pathways for modulating immunocompetence, skeletal, and gastrointestinal attributes in broiler chickens hatched from broiler breeders fed omega-3 polyunsaturated fatty acids.

The role of dietary polyunsaturated n-3 PUFA on immune modulation, microbiota regulation, and bone development has been explored individually. To the authors knowledge, this is the first time the role of omega-3 FA in the broiler breeder-broiler chickens interface regarding immune response, skeletal development and gut health has been reviewed. In general, there is consensus on the beneficial effects of reducing the n-6 PUFA to n-3 PUFA ratio by increasing dietary inclusion of n-3 PUFA. In this review, we examined the overall effects of n-3 PUFA on physiological and metabolic attributes in modern broilers. Majority of research characterizing the benefits of dietary n-3 PUFA examines the direct effects of feeding broiler chicks. However, there is an opportunity to apply concepts of developmental programming by utilizing broiler breeders to deposit n-3 FA in the yolk for embryonic utilization. While extensive research has been conducted on the feeding n-3 PUFA to BB and the subsequent effects on their progeny regarding their immune response, minimal data is available on the effect on progeny microbiota or skeletal development. There is an innate understanding that the immune status of a bird drives the health of the gut (microbiota) and plays a role in the signaling pathways involving bone health. However, quantitative, and qualitative relationship between these elements with respect to integrating n-3 PUFA provision in broiler diets and progeny outcomes is not well-defined (Figure 1). As a start, studies characterizing the effects of n-3 PUFA inclusion into BB diets on reproductive performance as measured by fertility, hatchability, and chick quality must be determined. Once these effects have been established, maternal feeding of n-3 PUFA may be examined in terms of subsequent effects on growth performance, immune status and bone development in broiler chickens. The review leads us to postulate the possibilities of mitigating some of the common physiological and metabolic issues seen in broiler chicks through maternal feeding of n-3 PUFA.

## Author Contributions

AT is a graduate student of EK, she searched literature and wrote significant portion of the review. EK overall conceptual and editorial responsibility.

## Conflict of Interest

The authors declare that the research was conducted in the absence of any commercial or financial relationships that could be construed as a potential conflict of interest.

## References

[1] Siegel PB. Evolution of the modern broiler and feed efficiency. Annu Rev Anim Biosci. (2014) 2:375–85. 10.1146/annurev-animal-022513-114132 25384148

[2] OECD/FAO. OECD - FAO Agricultural Outlook 2020–2029 (2020).

[3] Van Immerseel F Eeckhaut V Moore RJ Choct M Ducatelle R. Beneficial microbial signals from alternative feed ingredients: a way to improve sustainability of broiler production? Microb Biotechnol. (2017) 10:1008–11. 10.1111/1751-7915.12794 28840976 PMC5609280

[4] Julian RJ. Production and growth related disorders and other metabolic diseases of poultry-a review. Vet J. (2005) 169:350–69. 10.1016/j.tvjl.2004.04.015 15848778

[5] Cheema MA Qureshi MA Havenstein GB. A comparison of the immune response of a 2001 commercial broiler with a 1957 random bred broiler strain when fed representative 1957 and 2001 broiler diets. Poult Sci. (2003) 82:1519–29. 10.1093/ps/82.10.1519 14601727

[6] Kiarie E. Inherent digestive tract insufficiency in monogastric animals: culpability of the gut microbiome and dietary approaches for optimizing intestinal health (invited). In: Proceedings, Animal Nutrition Conference of Canada: Exploring the Links Between Animal Nutrition and Animal Health. Virtual (2020).

[7] Lemahieu C Bruneel C Termote-Verhalle R Muylaert K Buyse J Foubert I. Impact of feed supplementation with different omega-3 rich microalgae species on enrichment of eggs of laying hens. Food Chem. (2013) 141:4051–9. 10.1016/j.foodchem.2013.06.078 23993584

[8] Ao T Macalintal LM Paul MA Pescatore AJ Cantor AH Ford MJ. Effects of supplementing microalgae in laying hen diets on productive performance, fatty-acid profile, and oxidative stability of eggs. J Appl Poult Res. (2015) 24:394–400. 10.3382/japr/pfv042

[9] Akbari Moghaddam Kakhki R Ma DWL Price KR Moats JR Karrow NA Kiarie EG. Enriching ISA brown and Shaver white breeder diets with sources of n−3 polyunsaturated fatty acids increased embryonic utilization of docosahexaenoic acid. Poult Sci. (2020) 99:1038–51. 10.1016/j.psj.2019.09.002 32036961 PMC7587772

[10] Saber SN Kutlu HR. Effect of including n-3/n-6 fatty acid feed sources in diet on fertility and hatchability of broiler breeders and post-hatch performance and carcass parameters of progeny. Asian-Australas J Anim Sci. (2020) 33:305–12. 10.5713/ajas.19.0055 31010967 PMC6946961

[11] Williams B Solomon S Waddington D Thorp B Farquharson C. Skeletal development in the meat-type chicken. Brit Poult Sci. (2000) 41:141–9. 10.1080/713654918 10890208

[12] Butterworth A. Infectious components of broiler lameness: a review. World's Poult Sci J. (1999) 55:327–52. 10.1079/WPS19990024

[13] Williams B Waddington D Murray DH Farquharson C. Bone strength during growth: influence of growth rate on cortical porosity and mineralization. Calcif Tissue Int. (2004) 74:236–45. 10.1007/s00223-002-2124-0 14517713

[14] Wideman RF Jr. Bacterial chondronecrosis with osteomyelitis and lameness in broilers: a review. Poult Sci. (2016) 95:325–44. 10.3382/ps/pev320 26527707

[15] Ducatelle R Goossens E De Meyer F Eeckhaut V Antonissen G Haesebrouck F. Biomarkers for monitoring intestinal health in poultry: present status and future perspectives. Vet Res. (2018) 49:43. 10.1186/s13567-018-0538-6 29739469 PMC5941335

[16] Torok VA Hughes RJ Mikkelsen LL Perez-Maldonado R Balding K MacAlpine R. Identification and characterization of potential performance-related gut microbiotas in broiler chickens across various feeding trials. Appl Environ Microbiol. (2011) 77:5868–78. 10.1128/AEM.00165-11 21742925 PMC3165380

[17] Wilson HR. Effects of maternal nutrition on hatchability. Poult Sci. (1997) 76:134–43. 10.1093/ps/76.1.134 9037700

[18] Zuidhof MJ Fedorak MV Ouellette CA Wenger II. Precision feeding: innovative management of broiler breeder feed intake and flock uniformity. Poult Sci. (2017) 96 2254–63. 10.3382/ps/pex013 28159999

[19] Richards MP Rosebrough RW Coon CN McMurtry JP. Feed intake regulation for the female broiler breeder: in theory and in practice. J Appl Poult Res. (2010) 19:182–93. 10.3382/japr.2010-00167

[20] Inoue AY Berchieri A Bernardino A Paiva JB Sterzo EV. Passive immunity of progeny from broiler breeders vaccinated with oil-emulsion bacterin against salmonella enteritidis. Avian Diseases. (2008) 52:567–71, 565. 10.1637/8096-082707-Reg.1 19166046

[21] Poulsen LL Thofner I Bisgaard M Christensen JP Olsen RH Christensen H. Longitudinal study of transmission of Escherichia coli from broiler breeders to broilers. Vet Microbiol. (2017) 207:13–8. 10.1016/j.vetmic.2017.05.029 28757012

[22] Cox NA Richardson LJ Maurer JJ Berrang ME Fedorka-Cray PJ Buhr RJ. Evidence for horizontal and vertical transmission in Campylobacter passage from hen to her progeny. J Food Prot. (2012) 75:1896–902. 10.4315/0362-028.JFP-11-322 23043845

[23] Spratt RS Leeson S. Broiler breeder performance in response to diet protein and energy. Poult Sci. (1987) 66:683–93. 10.3382/ps.0660683 3615328

[24] Peebles ED Zumwalt CD Smith TW Gerard PD Latour MA. Poultry fat and corn oil may be used to adjust energy in the diets of young breeder hens without affecting embryogenesis and subsequent broiler growout performance. J Appl Poult Res. (2002) 11:146–54. 10.1093/japr/11.2.146

[25] Pearson RA Herron KM. Effects of energy and protein allowances during lay on the reproductive performance of broiler breeder hens. Bri Poult Sci. (1981) 22:227–39. 10.1080/00071688108447881

[26] Brenner RR. The desaturation step in the animal biosynthesis of polyunsaturated fatty acids. Lipids. (1971) 6:567–75. 10.1007/BF02531137 5094766

[27] Cherian G. Essential fatty acids and early life programming in meat-type birds. World's Poult Sci J. (2011) 67:599–614. 10.1017/S0043933911000705

[28] Simopoulos AP. The importance of the omega-6/omega-3 fatty acid ratio in cardiovascular disease and other chronic diseases. Exp Biol Med. (2008) 233:674–88. 10.3181/0711-MR-311 18408140

[29] Cherian G Sim J. Effect of feeding full fat flax and canola seeds to laying hens on the fatty acid composition of eggs, embryos, and newly hatched chicks. Poult Sci. (1991) 70:917–22. 10.3382/ps.0700917

[30] Thanabalan A Moats J Kiarie EG. Effects of feeding broiler breeder hens a coextruded full-fat flaxseed and pulses mixture without or with multienzyme supplement. Poult Sci. (2020) 99:2616–23. 10.1016/j.psj.2019.12.062 32359597 PMC7597534

[31] Noble R Cocchi M. Lipid metabolism and the neonatal chicken. Prog Lipid Res. (1990) 29:107–40. 10.1016/0163-7827(90)90014-C 2093896

[32] Kanakri K Carragher J Hughes R Muhlhausler B Gibson R. A reduced cost strategy for enriching chicken meat with omega-3 long chain polyunsaturated fatty acids using dietary flaxseed oil. Bri Poult Sci. (2017) 58:283–9. 10.1080/00071668.2017.1293798 28277795

[33] Lin DS Connor WE Anderson GJ. The incorporation of n-3 and n-6 essential fatty acids into the chick embryo from egg yolks having vastly different fatty acid compositions. Pediatric Research. (1991) 29:601–5. 10.1203/00006450-199106010-00015 1866216

[34] Liebler-Tenorio EM Pabst R. MALT structure and function in farm animals. Vet Res. (2006) 37:257–80. 10.1051/vetres:2006001 16611547

[35] Bar-Shira E Sklan D Friedman A. Establishment of immune competence in the avian GALT during the immediate post-hatch period. Dev Comp Immunol. (2003) 27:147–57. 10.1016/S0145-305X(02)00076-9 12543128

[36] Lammers A Wieland WH Kruijt L Jansma A Straetemans T Schots A. Successive immunoglobulin and cytokine expression in the small intestine of juvenile chicken. Dev Comp Immunol. (2010) 34:1254–62. 10.1016/j.dci.2010.07.001 20621117

[37] Lee KA. Linking immune defenses and life history at the levels of the individual and the species. Integr Comp Biol. (2006) 46:1000–15. 10.1093/icb/icl049 21672803

[38] Friedman A Elad O Choen I Shira EB. The gut associated lymphoid system in the post-hatch chick: dynamics of maternal IgA. Refu'ah veterinarit: riv'on Histadrut ha-rof'im ha-veterinariyim be-Erets-Yiśra'el. (2012) 67:75.

[39] Li S Zhi L Liu Y Shen J Liu L Yao J. Effect of *in ovo* feeding of folic acid on the folate metabolism, immune function and epigenetic modification of immune effector molecules of broiler. Br J Nutr. (2016) 115:411–21. 10.1017/S0007114515004511 26586196

[40] Korver DR Klasing KC. Dietary fish oil alters specific and inflammatory immune responses in chicks. J Nutr. (1997) 127:2039–46. 10.1093/jn/127.10.2039 9311962

[41] Calder P. N-3 polyunsaturated fatty acids and inflammation: from molecular biology to the clinic. Lipids. (2003) 38:343–52. 10.1007/s11745-003-1068-y 12848278 PMC7101988

[42] Calder P. n-3 fatty acids, inflammation and immunity-relevance to postsurgical and critically III patients. Lipids. (2004) 39:1147–61. 10.1007/s11745-004-1342-z 15736910 PMC7101959

[43] Kragballe K Voorhees JJ Goetzl EJ. Inhibition by leukotriene B5 of leukotriene B4-induced activation of human keratinocytes and neutrophils. J Invest Dermatol. (1987) 88:555–8. 10.1111/1523-1747.ep12470151 2437212

[44] Hall JA Jha S Skinner MM Cherian G. Maternal dietary n-3 fatty acids alter immune cell fatty acid composition and leukotriene production in growing chicks. Prostaglandins Leukot Essent Fatty Acids. (2007) 76:19–28. 10.1016/j.plefa.2006.09.003 17081738

[45] Koppenol A Delezie E Aerts J Willems E Wang Y Franssens L. Effect of the ratio of dietary n-3 fatty acids eicosapentaenoic acid and docosahexaenoic acid on broiler breeder performance, egg quality, and yolk fatty acid composition at different breeder ages. Poult Sci. (2014) 93:564–73. 10.3382/ps.2013-03320 24604849

[46] Cherian G Bautista-Ortega J Goeger DE. Maternal dietary n-3 fatty acids alter cardiac ventricle fatty acid composition, prostaglandin and thromboxane production in growing chicks. Prostaglandins Leukot Essent Fatty Acids. (2009) 80:297–303. 10.1016/j.plefa.2009.02.006 19442501

[47] Bullock CJ Bobe G Cherian G. Gastrointestinal and hepatic tissue fatty acidcomposition and interleukin-6 concentration in broiler chickens: effect of maternal dietary n-3 fatty acids. J Anim Sci. (2014) 92:414.24398845

[48] Wall R Ross RP Fitzgerald GF Stanton C. Fatty acids from fish: the anti-inflammatory potential of long-chain omega-3 fatty acids. Nutr Rev. (2010) 68:280–9. 10.1111/j.1753-4887.2010.00287.x 20500789

[49] Wang YW Field CJ Sim JS. Dietary polyunsaturated fatty acids alter lymphocyte subset proportion and proliferation, serum immunoglobulin G concentration, and immune tissue development in chicks. Poult Sci. (2000) 79:1741–8. 10.1093/ps/79.12.1741 11194036

[50] Bautista-Ortega J Goeger DE Cherian G. Egg yolk omega-6 and omega-3 fatty acids modify tissue lipid components, antioxidant status, and *ex vivo* eicosanoid production in chick cardiac tissue. Poult Sci. (2009) 88:1167–75. 10.3382/ps.2009-00027 19439626

[51] Butcher GG Mojtaba Y. Investigating Vaccination Failure in Poultry Flocks. Gainesville, FL (2008). Available online at: https://edis.ifas.ufl.edu/vm136#FOOTNOTE_1 (accessed at: *April 25, 2020*).

[52] Kapczynski DR Afonso CL Miller PJ. Immune responses of poultry to Newcastle disease virus. Dev Comp Immunol. (2013) 41:447–53. 10.1016/j.dci.2013.04.012 23623955

[53] Lu Z Thanabalan A Leung H Akbari Moghaddam KR Patterson R Kiarie EG. The effects of feeding yeast bioactives to broiler breeders and/or their offspring on growth performance, gut development, and immune function in broiler chickens challenged with Eimeria. Poult Sci. (2019) 98:6411–21. 10.3382/ps/pez479 31504867 PMC6870552

[54] Neijat M Habtewold J Shirley RB Welsher A Barton J Thiery P. Bacillus subtilis Strain DSM 29784 modulates the cecal microbiome, concentration of short-chain fatty acids, and apparent retention of dietary components in shaver white chickens during grower, developer, laying phases. Appl Environ Microbiol. (2019) 85:e00402–19. 10.1128/AEM.00402-19 31076425 PMC6606875

[55] Rubio LA. Possibilities of early life programming in broiler chickens via intestinal microbiota modulation. Poult Sci. (2019) 98:695–706. 10.3382/ps/pey416 30247675

[56] Alagawany M Elnesr SS Farag MR Abd El-Hack ME Khafaga AF Taha AE. Omega-3 and omega-6 fatty acids in poultry nutrition: effect on production performance and health. Animals. (2019) 9:573. 10.3390/ani9080573 31426600 PMC6721126

[57] Guo Y Chen S Xia Z Yuan J. Effects of different types of polyunsaturated fatty acids on immune function and PGE2 synthesis by peripheral blood leukocytes of laying hens. Ani Feed Sci Technol. (2004) 116:249–58. 10.1016/j.anifeedsci.2004.07.011

[58] Akbari Moghaddam Kakhki R Ma DWL Price KR Moats J Karrow NA Kiarie EG. Impact of feeding n-3 fatty acids to layer breeders and their offspring on concentration of antibody titers against infectious bronchitis, and Newcastle diseases and plasma fatty acids in the offspring. Brit Poult Sci. (2020) 62:270–7. 10.1080/00071668.2020.1847254 33155822

[59] Korver DR Wakenell P Klasing KC. Dietary fish oil or lofrin, a 5-lipoxygenase inhibitor, decrease the growth-suppressing effects of coccidiosis in broiler chicks. Poult Sci. (1997) 76:1355–63. 10.1093/ps/76.10.1355 9316110

[60] Maki JJ Klima CL Sylte MJ Looft T. The microbial pecking order: utilization of intestinal microbiota for poultry health. Microorganisms. (2019) 7:376. 10.3390/microorganisms7100376 31547217 PMC6843665

[61] Awad WA Hess C Hess M. Enteric pathogens and their toxin-induced disruption of the intestinal barrier through alteration of tight junctions in chickens. Toxins. (2017) 9:60. 10.3390/toxins9020060 28208612 PMC5331439

[62] Chen ML Ge Z Fox JG Schauer DB. Disruption of tight junctions and induction of proinflammatory cytokine responses in colonic epithelial cells by *Campylobacter jejuni*. Infect Immun. (2006) 74:6581–9. 10.1128/IAI.00958-06 17015453 PMC1698078

[63] Kiarie E Romero LF Nyachoti CM. The role of added feed enzymes in promoting gut health in swine and poultry. Nutr Res Rev. (2013) 26:71–88. 10.1017/S0954422413000048 23639548

[64] Brisbin JT Gong J Sharif S. Interactions between commensal bacteria and the gut-associated immune system of the chicken. Anim Health Res Rev. (2008) 9:101–10. 10.1017/S146625230800145X 18541076

[65] Huyghebaert G Ducatelle R Immerseel FV. An update on alternatives to antimicrobial growth promoters for broilers. Vet J. (2011) 187:182–8. 10.1016/j.tvjl.2010.03.003 20382054

[66] Teirlynck E Gussem MD Dewulf J Haesebrouck F Ducatelle R Van Immerseel F. Morphometric evaluation of “dysbacteriosis” in broilers. Avian Pathol. (2011) 40:139–44. 10.1080/03079457.2010.543414 21500033

[67] Stanley D Geier MS Hughes RJ Denman SE Moore RJ. Highly variable microbiota development in the chicken gastrointestinal tract. PLoS ONE. (2013) 8:e84290. 10.1371/journal.pone.0084290 24391931 PMC3877270

[68] Ballou AL Ali RA Mendoza MA Ellis JC Hassan HM Croom WJ. Development of the chick microbiome: how early exposure influences future microbial diversity. Front Vet Sci. (2016) 3:2. 10.3389/fvets.2016.00002 26835461 PMC4718982

[69] Lee S La TM Lee HJ Choi IS Song CS Park SY. Characterization of microbial communities in the chicken oviduct and the origin of chicken embryo gut microbiota. Sci Rep. (2019) 9:6838. 10.1038/s41598-019-43280-w 31048728 PMC6497628

[70] Torok VA Allison GE Percy NJ Ophel-Keller K Hughes RJ. Influence of antimicrobial feed additives on broiler commensal posthatch gut microbiota development and performance. Appl Environ Microbiol. (2011) 77:3380–90. 10.1128/AEM.02300-10 21441326 PMC3126468

[71] Kiarie EG Mills A. Role of feed processing on gut health and function in pigs and poultry: conundrum of optimal particle size and hydrothermal regimens. Front Vet Sci. (2019) 6:19. 10.3389/fvets.2019.00019 30838217 PMC6390496

[72] Cândido FG Valente FX Grześkowiak ŁM Moreira APB Rocha DMUP Alfenas RCG. Impact of dietary fat on gut microbiota and low-grade systemic inflammation: mechanisms and clinical implications on obesity. Int J Food Sci Nutr. (2018) 69:125–43. 10.1080/09637486.2017.1343286 28675945

[73] Geier MS Torok VA Allison GE Ophel-Keller K Gibson RA Munday C. Dietary omega-3 polyunsaturated fatty acid does not influence the intestinal microbial communities of broiler chickens. Poult Sci. (2009) 88:2399–405. 10.3382/ps.2009-00126 19834092

[74] Azad AB Sarker M Li T Yin J. Probiotic species in the modulation of gut microbiota: an overview. Biomed Res Int. (2018) 2018:9478630. 10.1155/2018/9478630 29854813 PMC5964481

[75] Oakley BB Kogut MH. Spatial and temporal changes in the broiler chicken cecal and fecal microbiomes and correlations of bacterial taxa with cytokine gene expression. Front Vet Sci. (2016) 3:11. 10.3389/fvets.2016.00011 26925404 PMC4759570

[76] Rychlik I. Composition and function of chicken gut microbiota. Animals. (2020) 10:103. 10.3390/ani10010103 31936291 PMC7022619

[77] Robertson RC Kaliannan K Strain CR Ross RP Stanton C Kang JX. Maternal omega-3 fatty acids regulate offspring obesity through persistent modulation of gut microbiota. Microbiome. (2018) 6:95. 10.1186/s40168-018-0476-6 29793531 PMC5968592

[78] Kaliannan K Wang B Li XY Kim KJ Kang JX. A host-microbiome interaction mediates the opposing effects of omega-6 and omega-3 fatty acids on metabolic endotoxemia. Sci Rep. (2015) 5:11276. 10.1038/srep11276 26062993 PMC4650612

[79] Bradshaw RH Kirkden RD Broom D. A review of the aetiology and pathology of leg weakness in broilers in relation to welfare. Avian Poult Biol Rev. (2002) 13:45–103. 10.3184/147020602783698421

[80] Whitehead CC Fleming RH. Osteoporosis in cage layers. Poult Sci. (2000) 79:1033–41. 10.1093/ps/79.7.1033 10901207

[81] Fong L Muhlhausler BS Gibson RA Xian CJ. Perinatal maternal dietary supplementation of ω3-fatty acids transiently affects bone marrow microenvironment, osteoblast and osteoclast formation, and bone mass in male offspring. Endocrinology. (2012) 153:2455–65. 10.1210/en.2011-1917 22374977

[82] Lau BY Cohen DJ Ward WE Ma DW. Investigating the role of polyunsaturated fatty acids in bone development using animal models. Molecules. (2013) 18:14203–27. 10.3390/molecules181114203 24248147 PMC6270577

[83] Uni Z Ferket P Tako E Kedar O. *In ovo* feeding improves energy status of late-term chicken embryos. Poult Sci. (2005) 84:764–70. 10.1093/ps/84.5.764 15913189

[84] Moran ET Jr. Nutrition of the developing embryo and hatchling. Poult Sci. (2007) 86:1043–9. 10.1093/ps/86.5.1043 17435045

[85] Lau BY Ward WF Kang JX Kang JF Ma DWL Ma DW. Vertebrae of developing fat-1 mice have greater strength and lower n-6/n-3 fatty acid ratio. Exp Biol Med. (2009) 234:632–8. 10.3181/0808-RM-247 19307460

[86] Watkins BA Shen CL McMurtry JP Xu H Bain SD. Dietary lipids modulate bone prostaglandin e2 production, insulin-like growth factor-i concentration and formation rate in chicks. J Nutr. (1997) 127:1084–91. 10.1093/jn/127.6.1084 9187621

[87] Li Y Greiner RS Salem N Watkins BA. Impact of dietary n-3 FA deficiency on rat bone tissue FA composition. Lipids. (2003) 38:683–6. 10.1007/s11745-003-1115-8 12934680

[88] Lau BY Fajardo VA McMeekin L Sacco SM Ward WE Roy BD. Influence of high-fat diet from differential dietary sources on bone mineral density, bone strength, and bone fatty acid composition in rats. Appl Physiol Nutr Metab. (2010) 35:598–606. 10.1139/H10-052 20962915

[89] Li Y Seifert MF Lim SY Salem N Watkins BA. Bone mineral content is positively correlated to n-3 fatty acids in the femur of growing rats. Br J Nutr. (2010) 104:674–85. 10.1017/S0007114510001133 20420751

[90] Lukas R Gigliotti JC Smith BJ Altman S Tou JC. Consumption of different sources of omega-3 polyunsaturated fatty acids by growing female rats affects long bone mass and microarchitecture. Bone. (2011) 49:455–62. 10.1016/j.bone.2011.05.029 21672645

[91] Liu D Veit HP Wilson JH Denbow DM. Long-term supplementation of various dietary lipids alters bone mineral content, mechanical properties and histological characteristics of Japanese quail. Poult Sci. (2003) 82:831–9. 10.1093/ps/82.5.831 12762407

[92] Mollard RC Gillam ME Wood TM Taylor CG Weiler HA. (n-3) Fatty acids reduce the release of prostaglandin e2 from bone but do not affect bone mass in obese (fa/fa) and lean zucker rats. J Nutr. (2005) 135:499–504. 10.1093/jn/135.3.499 15735084

[93] Baird HT Eggett DL Fullmer S. Varying ratios of omega-6: omega-3 fatty acids on the pre-and postmortem bone mineral density, bone ash, and bone breaking strength of laying chickens. Poult Sci. (2008) 87:323–8. 10.3382/ps.2007-00186 18212376

[94] Green KH Wong SCF Weiler HA. The effect of dietary n-3 long-chain polyunsaturated fatty acids on femur mineral density and biomarkers of bone metabolism in healthy, diabetic and dietary-restricted growing rats. Prostagl Leukot Essent Fatty Acids. (2004) 71:121–30. 10.1016/j.plefa.2004.03.001 15207529

[95] Tarlton JF Wilkins LJ Toscano MJ Avery NC Knott L. Reduced bone breakage and increased bone strength in free range laying hens fed omega-3 polyunsaturated fatty acid supplemented diets. Bone. (2013) 52:578–86. 10.1016/j.bone.2012.11.003 23142806

[96] Ebeid T Fayoud A El-Soud SA Eid Y El-Habbak M. The effect of omega-3 enriched meat production on lipid peroxidation, antioxidative status, immune response and tibia bone characteristics in Japanese quail. Czech J Anim Sci. (2011) 56:314–24. 10.17221/1293-CJAS

[97] Koren N Simsa-Maziel S Shahar R Schwartz B Monsonego-Ornan E. Exposure to omega-3 fatty acids at early age accelerate bone growth and improve bone quality. J Nutr Biochem. (2014) 25:623–33. 10.1016/j.jnutbio.2014.01.012 24746838

[98] Akbari Moghaddam Kakhki R Price KR Moats J Bédécarrats G Karrow NA Kiarie EG. Impact of feeding microalgae (*Aurantiochytrium limacinum*) and co-extruded mixture of full-fat flaxseed as sources of n-3 fatty acids to ISA brown and Shaver white breeders and progeny on pullet skeletal attributes at hatch through to 18 weeks of age. Poult Sci. (2020) 99:2087–99. 10.1016/j.psj.2019.12.016 32241494 PMC7587698

[99] Kruger MC Coetzee M Haag M Weiler H. Long-chain polyunsaturated fatty acids: selected mechanisms of action on bone. Progr. Lipid Res. (2010) 49:438–49. 10.1016/j.plipres.2010.06.002 20600307

[100] Atkinson TG Barker HJ Meckling-Gill KA. Incorporation of long-chain n-3 fatty acids in tissues and enhanced bone marrow cellularity with docosahexaenoic acid feeding in post-weanling Fischer 344 rats. Lipids. (1997) 32:293–302. 10.1007/s11745-997-0036-x 9076666

[101] Shen CL Yeh JK Rasty J Li Y Watkins BA. Protective effect of dietary long-chain n-3 polyunsaturated fatty acids on bone loss in gonad-intact middle-aged male rats. Br J Nutr. (2006) 95:462–8. 10.1079/BJN20051664 16512931

[102] Kajarabille N Díaz-Castro J Hijano S López-Frías M López-Aliaga I Ochoa JJ. A new insight to bone turnover: role of omega-3 polyunsaturated fatty acids. Sci World J. (2013) 2013:589641. 10.1155/2013/589641 24302863 PMC3834626

[103] Shen CL Peterson J Tatum OL Dunn DM. Effect of long-chain n-3 polyunsaturated fatty acid on inflammation mediators during osteoblastogenesis. J Med Food. (2008) 11:105–10. 10.1089/jmf.2007.540 18361745

[104] Watkins BA Li Y Allen KGD Hoffmann WE Seifert MF. Dietary Ratio of (n-6)/(n-3) Polyunsaturated fatty acids alters the fatty acid composition of bone compartments and biomarkers of bone formation in rats. J Nutr. (2000) 130:2274–84. 10.1093/jn/130.9.2274 10958824

[105] Tong X Zhang J Li J. LPS-induced inflammation disorders bone modeling and remodeling by inhibiting angiogenesis and disordering osteogenesis in chickens. Inflamm Res. (2020) 69:765–77. 10.1007/s00011-020-01361-x 32444883

